# Differentiation of Andean and Mesoamerican accessions in a proposed core collection of grain amaranths

**DOI:** 10.3389/fpls.2023.1144681

**Published:** 2023-03-22

**Authors:** Matthew W. Blair, Jorge M. Londoño, María A. Buitrago-Bitar, Xingbo Wu, David M. Brenner

**Affiliations:** ^1^ Department of Agricultural and Environmental Sciences, College of Agriculture, Tennessee State University, Nashville, TN, United States; ^2^ Grupo de Investigación en Biodiversidad y Biotecnología (GIBUQ), Universidad del Quindío, Armenia, Colombia; ^3^ Facultad de Ciencias Agropecuarias, Universidad Nacional de Colombia, Palmira, Colombia; ^4^ Tropical Research Education Center, University of Florida, Homestead, FL, United States; ^5^ Department of Agronomy/North Central Regional Plant Introduction Station, Iowa State University, Ames, IA, United States

**Keywords:** Andean grain amaranth, cultivated and wild accessions, genetic diversity, Mesoamerican grain amaranth, centers of domestication, competitive allele specific PCR

## Abstract

Grain amaranths are made up of three New World species of pseudo-cereals with C_4_ photosynthesis from the dicotyledonous family Amaranthaceae and the genus *Amaranthus*. They originate in two ecoregions of the Americas, namely, the inter-Andean valleys of South America and the volcanic axis and lowlands of Mexico and Central America. These correspond to two centers of domestications for Andean and Mesoamerican crops, with one cultivated species found in the first region and two found in the latter region. To date, no core collection has been made for the grain amaranths in the United States Department of Agriculture (USDA) germplasm system. In this study, our objective was to create a core for the 2,899 gene bank accessions with collection site data by town or farm site of which 1,090 have current geo-referencing of latitude and longitude coordinates. We constituted the core with 260 genotypes of *Amaranthus*, which we evaluated with 90 single-nucleotide polymorphism markers. Our goal was to distinguish between Andean and Mesoamerican gene pools of amaranths, including the cultivated species and three possible progenitor or wild relative ancestors along with two more species in an outgroup. Population structure, clustering, and discriminant analysis for principal components showed that Andean species *Amaranthus caudatus* and *Amaranthus quitensis* shared fewer alleles with Mesoamerican species *Amaranthus cruentus* and *Amaranthus hypochondriacus*, compared to each group individually. *Amaranthus hybridus* was a bridge species that shared alleles with both regions. Molecular markers have the advantage over morphological traits at quickly distinguishing the Andean and Mesoamerican cultivars and have the added benefit of being useful for following inter-species crosses and introgression.

## Introduction

Core collections (CCs) are an especially popular form of subsampling plant gene banks (Brown, 1989). This is the case with genetic resources conserved as seeds or asexual propagules, but especially for grain species which are abundantly collected by governments for food security and sovereignty, A “core” is usually made by a gene bank germplasm curator based on phenotypic trait characterization or DNA analysis of accessions (van Hintum et al., 2000). Usually, 10% of the total germplasm is sampled, with this reduced in the case of very large collections (e.g., Oliveira et al., 2010). Smaller collections such as those for orphan species such as quinoa (Ortiz et al., 1998) or millets (Upadhyaya et al., 2011a; Upadhyaya et al., 2011b) have fewer accessions in them. The goal of the CC is to display the diversity found in a gene bank for a given species (Schafleitner et al., 2022). CCs tend to be from only one institution at a time rather than across them (van Hintum et al., 2000). Major gene banks for crop plants include the Plant Genetic Resources System of the United States Department of Agriculture (USDA) and the network of Genetic Resources Units in the Consultative Group for International Agriculture (CGIAR). Non-crop plants are usually conserved in private collections, while commodities are guarded at the national level for biodiversity-rich countries. Fortunately, some duplication of accessions between gene banks, often in the form of backup seed, allows comparisons of CCs from one institution to another.

Grain amaranths are a group of three species of promising pseudo-cereal crops with light-colored but tiny-sized seeds, which were domesticated in two regions of the Americas by the pre-colonized, indigenous peoples of North and South America (Riggins et al., 2021). The species include *Amaranthus caudatus* (South American) and *Amaranthus cruentus* and *Amaranthus hypochondriacus* (Mesoamerican/North American), but all go under the same common name of grain amaranth in English. For the most part, they are called “amaranto” in Spanish. Each cultivated species originated from weedy species in the same genus (Blair et al., 2022). They conserve indigenous names of “kiwicha” for the South American species and “huautli” for the North American ones. Among the two Mesoamerican species, the basic chromosome number is n = 16, while the Andean species has n = 17, although all three are true diploids (Andini et al., 2018).

They also share characteristics of being monoecious, adaptable to a wide range of environments, with high levels of drought/salinity tolerance and C_4_ photosynthesis (Dharajiya et al., 2022; Jamalluddin et al., 2022). The South American species was domesticated near the equator and is much more photoperiod sensitive than the North American species, which from here on in we will refer to as Mesoamerican. Conservation of grain amaranths falls primarily to national programs in the centers of origin, followed by the USDA collection (Ames, Iowa) and finally a subset of the mostly vegetable-type collection in WorldVeg (Hoshikawa et al., 2023).

Genetic resources for a species can be divided into those that are *in vitro* (seed bank) accessions versus *in vivo* (on farm or natural) genotypes. *In vivo* genotypes can be landraces, farmers’ varieties, or weeds and escapes. In contrast, weed amaranths tend to be majority outcrossing, and grain amaranths tend to be self-pollinating especially when managed in gene banks. Therefore, it was important to use *in vitro* genotypes for our study because they have been purified and divided into grain amaranth species by a gene bank curator. Currently, USDA has 2,899 such *Amaranthus* with collection data. Our CC samples approximately 10% of these.

The *in vitro* accessions were also easier to obtain, as they are quite prolific in seed production even in USDA greenhouse conditions where they are likely to be inbred. In this study, we used only *in vitro* and cultivated type grain amaranths from a proposed USDA core collection tested in temperate environments (Thapa and Blair, 2019). Our goal was to determine within and between species diversity for the three species making up this crop. We hypothesized from previous genetic analysis (Thapa et al., 2021) that grain amaranths could be divided into Andean species (cultivated *A. caudatus* and weedy relative, *Amaranthus quitensis*) versus Mesoamerican species (*A. cruentus* and *A. hypochondriacus*). This hypothesis was tested in the current study using molecular markers based on competitive allele single-nucleotide polymorphism (SNP) markers.

## Materials and methods

### Plant materials

Seeds were sourced from the USDA, North Central Regional Plant Introduction Station at Iowa State University for grain amaranths conserved in Ames, Iowa, USA. The proposed CC for grain amaranths studied here included 260 genotypes (**Table S1**), approximately 10% of USDA-collected *Amaranthus*. We had grown these same entries outdoors in Nashville, TN, and in a greenhouse at Tennessee State University (TSU) previously, so we knew them to be grain amaranths or close relatives (Thapa and Blair, 2019). Our CC included 199 accessions from cultivated species and 61 wild accessions. In addition, the CC was weighted toward three cultivated species: 1) *A. cruentus* (120 genotypes), 2) *A. hypochondriacus* (44), and 3) *A. caudatus* (33). Apart from the cultivars, there were representatives of five wild or weedy species: *Amaranthus hybridus* (24), *A. quitensis* (18), *Amaranthus powellii* (6), *Amaranthus retroflexus* (2), and *Amaranthus palmeri* (1). A total of 11 entries in the CC had undefined species identification (PI262395, PI527567, PI584523, PI594692, PI604461, PI604576, PI604577, PI619237, PI619239, PI643045, and PI643056). The CC accessions are maintained by the USDA National Plant Germplasm System station in Ames, Iowa, in greenhouses and under tenting to prevent outcrossing. DNA was extracted from all these genotypes using *in vitro* germinated seedling tissue as described in Thapa et al. (2021). Briefly, genomic DNA of each accession was extracted from the leaf and stem of small seedlings from Magenta box agar tissue culture using FASTDNA^®^ miniprep kits (MP Biomedical, Solon, OH, USA) quantified with a NanoDrop 1000 UV-Vis Spectrophotometer (Thermo Fisher Scientific Inc., Waltham, MA, USA) and diluted with autoclaved ultrapure water to prepare working stocks of 10 ng/μl before PCR amplification with the methods described below.

### SNP markers utilized

The genetic markers we used consisted of KASP assays targeting 90 separate loci, with 45 having been used originally in Thapa et al. (2021) and 45 newly added ones, all with high polymorphism information content according to their developer (Maughan et al., 2009). A total of 82 amplified well for genetic analysis (**Table S1**). All these markers had been developed previously for *A. caudatus* and tested across a range of species by Maughan et al. (2011). However, eight markers from the two groups were non-amplifiable including AM19583 from the previous study and the following markers in this study: AM19425, AM19663, AM19811, AM20090, AM20720, AM21120, and AM21743. All amplified markers from both sets were bi-allelic. The KASP marker primer sets and master mix were ordered from LGC Inc. (Beverly, MA, USA). Each assay consisted of three oligonucleotides targeting the SNP locus to detect alternative nucleotides for each bi-allelic option. PCR conditions, reaction mixes, and plate sealing were as described previously in Thapa et al. (2021) with a few modifications for high throughput analysis. The modifications consisted of the following. First, amplification was carried out in MicroAmp™ EnduraPlate™ Optical 384-Well Clear Reaction Plates from Fisher Scientific instead of 96-well plates on a Hydrocycler (LGC Genomics Ltd., Sheffield, UK). PCR conditions were as follows: 10 cycles of denaturation at 92°C for 30 s, extension-annealing at 65°C for 1 min, followed by 26 cycles of 92°C for 30 s, and 57°C extension-annealing for 1 min. Second, the total reaction volume was reduced to 6 µl from 10 µl with only 1 µl (10 ng) of template DNA combined with 1 µl of the three-oligonucleotide mixture and 0.6 µl of the enzyme/master mix provided by LGC Inc. Subsequently, the 388 well plate reactions were placed in the exposure cabinet of a FLUOstar Omega fluorescence reader (BMG Labtech Inc., Cary, NC, USA) and scanned using excitation fluorometry.

### Data analysis

Data were collected for the KASP assays of all individual markers based on emission wavelengths of 520 nm (for FAM), 556 nm (HEX), and 610 nm (ROX). After this fluorescent scanning step, the data were interpreted using KlusterCaller software (LGC). The results were translated into a data matrix of nucleotides at each SNP locus for each genotype. These allele calls were copied into an MS Excel file for further analyses, which included the following: in the first step, major allele frequency (MAF), genetic diversity (GD), observed heterozygosity (Ho), and polymorphism information content (PIC) for each KASP marker were estimated using the software program, Power Marker v. 3.25 (https://brcwebportal.cos.ncsu.edu/powermarker/), developed by Liu and Muse (2005).

In a second step, population structure analysis was carried out using the software program STRUCTURE v.2.3.3 developed by Pritchard et al. (2000). The program was run with *a priori* genotype assignments into species. Only five species with more than n = 10 genotypes were included, but the hypotheses of two to eight populations were tested. The number of populations (K) was evaluated with 50,000 burn-ins and 50,000 Markov chain Monte Carlo (MCMC) iterations with 10 repetitions. The ideal population number was found with Structure Harvester software (https://taylor0.biology.ucla.edu/structureHarvester/faq.html) developed by Earl and von Holdt (2012) and graphed with R package *pophelper* from Francis (Francis, 2017). An individual with a threshold value of more than 85% genome fraction was assigned to a population.

In a third step, phylogenetic analysis was conducted using the software program Molecular Evolution Genetics Analysis or MEGA v.11 (https://www.megasoftware.net/) using the Tamura 92 model with a posterior probability of 1,000 bootstrapping replicates (Tamura et al., 2021). The maximum likelihood dendrogram was drawn in a circular manner for a more precise study of relationships between accessions and species. In this case, *A. powellii* was used as an outgroup, and accessions could be divided into five other groups based on the dendrogram. In the fourth step, analyses of molecular variance (AMOVAs) were performed comparing species (population level) and center of origins (subpopulations), and corresponding boxplot and clustered heatmap for pairwise F_ST_ values were calculated in R packages *poppr* and *pegas-ape*, respectively. Finally, a discriminant analysis for principal components (DAPC) was performed in three dimensions to observe the separation of species using package *adegenet* (Jombart et al., 2010) in the same R software. GeneAlex v.6.5 was used for a principal component analysis (PCA) in three dimensions, visualized with BioVinci (https://www.biostars.org/p/316074/) program to better differentiate the *Amaranthus* species analyzed.

## Results

### SNP marker success

A total of 90 KASP markers were used for the analysis of the grain amaranth CC with 92% of them showing easy-to-read polymorphism. In total, 45 were new ones apart from the set of 45 run by Thapa et al. (2021). Of these, 38 SNP assays were amplifiable and polymorphic, together with the 44 polymorphic markers from the previous study that provided a total of 82 markers for the diversity analysis of the USDA core collection. In total, 20,008 data points were generated for the 244 genotypes kept from the 260 DNAs first included. All these genotype × marker combinations amplified and were polymorphic.

Diversity values were estimated for each of the KASP markers and were provided for both newly run markers and those from Thapa et al. (2021) for ease of comparison (**Table S1**). MAF ranged from 0.5 to 0.95 for the 82 AM markers tested and averaged 0.73. GD for the markers ranged from 0.08 (for AM19963) to AM24210 (0.65). However, most markers had GD values in a narrower band from 0.24 to 0.45. Other markers with high GD values included AM27642 (0.63), AM21859 (0.61), and AM19855 (0.60). AM25418 and AM25418 had low GD values of 0.09 and 0.11, respectively. Overall, the average GD value was 0.38. Observed heterozygosity was low except for markers AM19210, AM19378, AM19426, AM22649, and AM27636. The average observed heterozygosity across all marker × genotype combinations was 0.09. PIC values for the markers were found to average 0.31. The range in PIC values was from 0.09 for marker AM25418 to 0.57 for marker AM24210.

### Population structure of accessions

**Figure 1:**
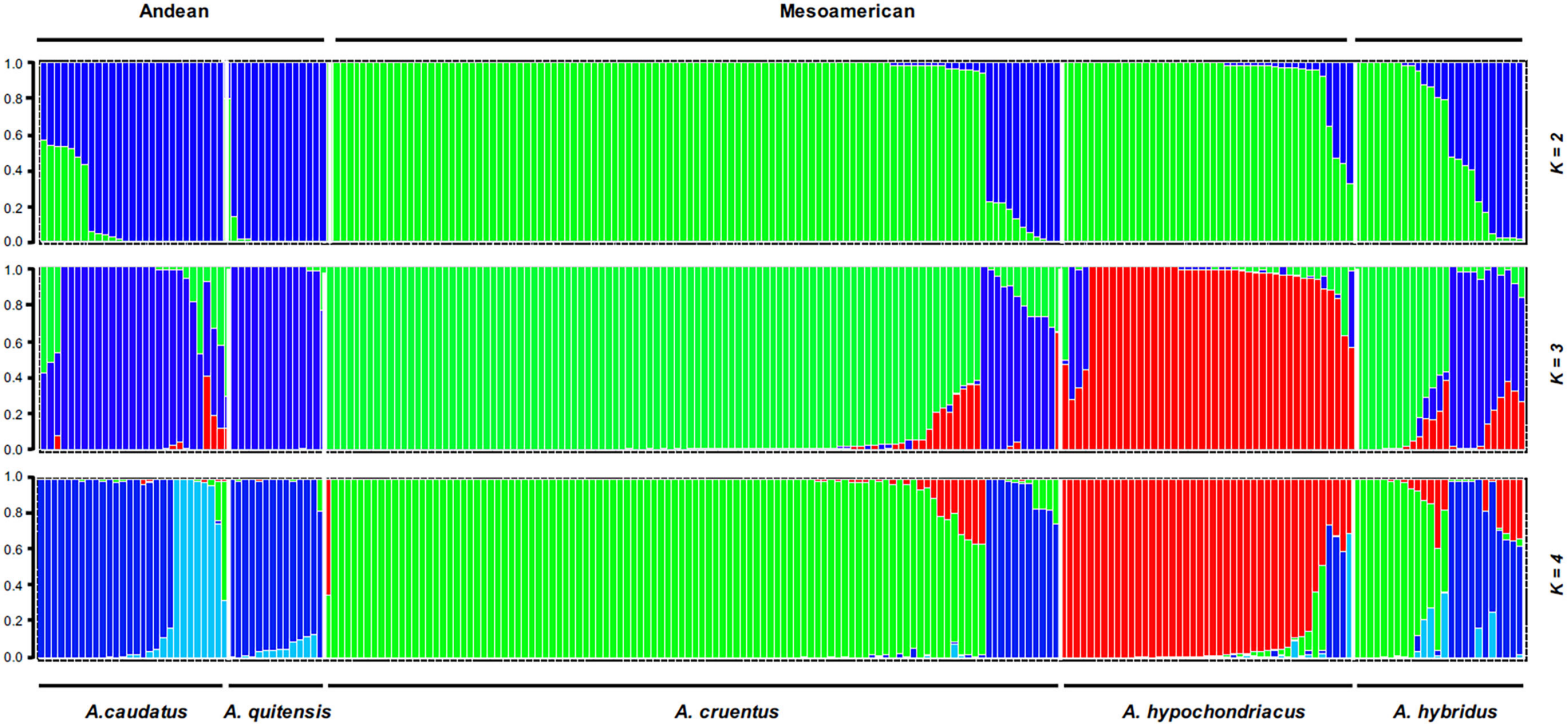
Population structure for K = 2 to K = 4 clusters with grain amaranth accessions (*Amaranthus caudatus*, *Amaranthus cruentus*, and *Amaranthus hypochondriacus*) and wild relatives (*Amaranthus quitensis* and *Amaranthus hybridus*) divided by species with region of origin (Andean *versus* Mesoamerican and mixed) shown above the bars.

Population structure analysis was performed for the three grain amaranth species (*A. caudatus*, *A. cruentus*, and *A. hypochondriacus*) and their immediate weedy precursors (*A. quitensis* and *A. hybridus*) at K values up to 8. The ideal number of populations was found to be K = 3 based on the Evanno tests in Structure Harvester, but both K = 2 and K = 4 are diagramed (**Figure 1**), as each level of structure in this range was informative. At K = 2, the two South American species, one cultivated and one wild (*A. caudatus* and *A. quitensis*, respectively), were grouped in the same color (blue), indicating population identity. This population will be interchangeably termed the Andean group, as it is from the Andes Mountains.

Meanwhile, the two North American cultivated species (*A. cruentus* and *A. hypochondriacus*) were grouped in a different color (green). This group will be termed the Mesoamerican group, as it is from a region broadly defined as Central America and Mexico within the sub-tropical parts of North America.

Interestingly, the cross-continental wild species, *A. hybridus*, found as a weed and natural species across most of the Americas (New World species) was divided into both blue and green colors with intermediate genotypes and high amounts of admixture (Q = 0.5 to 0.85) for many accessions, suggesting it as an origin species for both the Andean and Mesoamerican groups and also very diverse with its own range of diversity broader than for the other *Amaranthus* spp.

Color coding at K = 3 showed that the third color (red) now represented *A. hypochondriacus* species alone, divided off from the Mesoamerican group. The *A. hybridus* group had admixture again at this K-level. The highest probability ΔK value was at this level of structure, showing the separation of all three species of cultivated grain amaranths to be robustly supported. The K = 4 is shown because some subdivision of the *A. quitensis* and *A. caudatus* grouping was seen. In summary, the two wild species were grouped in different ways with the cultivars: *A. quitensis* shared ancestry with *A. caudatus* predominantly and with the other species partially. Meanwhile, *A. hybridus* shared ancestry with all three cultivated species and even with *A. quitensis*.

### Analysis of molecular variance

With the hypothesis of the Andean and Mesoamerican grouping of the cultivated accessions partly resolved by population structure, we wanted to continue our analysis with an AMOVA to determine species boundaries among cultivated and wild species and the possible importance of subpopulations based on the continent of origin. To do this, we prepared a stratified hierarchical AMOVA using R statistical software package. First, all samples were categorized at “population” and “subpopulation” levels. The population category was based on species classification from USDA. Since there were eight species, there were an equal number of populations.

The subpopulation category was based on assignments within each population using the origin of the accessions at the six regional or continental levels (Africa, Asia, Europe, North/Central America, and South America). The independent and combinational effects (subpopulation within populations) were evaluated, and in each case, sum of squares (SSq), mean squares (MSq), and percentage variation (%) explained were calculated (**Table 1**).

**Table 1:** Analysis of molecular variance considering population and subpopulations found in structure analysis of the core collection of grain amaranths and their wild relatives.

All levels independently					
	*Df*	Sum Sq	Mean Sq	Sigma	%
**Between Populations**	4	4,507.1047	1,126.77618	13.859062	38.94
**Between subpop**	16	906.1795	56.63622	1.691292	4.75
**Between samples**	197	6,431.2249	32.64581	12.605016	35.41
**Within samples**	218	1,621.0000	7.43578	7.435780	20.89
**Total**	435	13,465.5092	30.95519	35.591150	100.00
Population levels independently					
**Between populations**	4	4,507.105	1,126.77618	14.67658	41.21
**Between samples**	213	7,337.404	34.44791	13.50606	37.92
**Within samples**	218	1621	7.43578	7.43578	20.88
**Total**	435	13,465.509	30.95519	35.61843	100.00
Subpopulation levels independently					
**Between subpop**	6	2,818.239	469.70652	7.61249	23.26
**Between samples**	211	9,026.27	42.77853	17.67138	54.01
**Within samples**	218	1621	7.43578	7.43578	22.72
**Total**	435	13,465.509	30.95519	32.71965	100.00

AMOVA results with all levels analyzed together showed that species-level (population) differences were more important (38.94% of variation) compared to continental differences within species or between subpopulation differences (4.75%) with large amounts of variation both between and within samples. With only species analyzed, the percentage went up to 41%, while when only continents were analyzed, it was 23%. Differences between South and North America could make up most of this distinction.

### Pairwise F_ST_ values for species

Fixation indices were determined for each species compared to the total genetic variance detected. **Table 2** shows the pairwise F_ST_ values for the species comparisons. Values ranged from 0.04 between Andeans *A. caudatus* and *A. quitensis* and up to 0.42 between Mesoamerican *A. hypochondriacus* and Andean *A. quitensis*. The values were also high comparing Mesoamerican *A. hypochondriacus* to Andean *A. caudatus* (0.38).

**Table 2:** Pairwise F_ST_ values among species from the core collection for grain amaranths and wild relatives.

	No.	Amaranthus quitensis	Amaranthus cruentus	Amaranthus hypochondriacus	Amaranthus hybridus	Amaranthus powellii
***A. caudatus***	33	0.04774821	0.1931901	0.37758664	0.13844556	0.22340991
***A. quitensis***	18	0	0.21133041	0.42445988	0.22371884	0.40430709
***A. cruentus***	120	–	0	0.24377767	0.05751556	0.06006204
***A. hypochondriacus***	44	–	–	0	0.22536694	0.16951500
***A. hybridus***	24	–	–	–	0	0.16435465
***A. powellii***	6	–	–	–	–	0

Comparisons of *A. cruentus* and possible progenitor *A. hybridus* to the two Andean species had intermediate F_ST_ values (0.13 to 0.22). *A. powellii* was more distinct from both Andeans and from the one Mesoamerican species *A. hypochondriacus* but shared similarities with the other Mesoamerican species, *A. cruentus*. Overall, moderate differentiation of species can be inferred based on the highest possible F_ST_ value being 1.0. The F_ST_ values between the cultivated grain amaranths and their wild relatives with the true weed species, *A. palmeri* (n = 1) and *A. retroflexus* (n = 2), were high ranging up to 0.70 (data not shown). However, SNP genotyping with fewer accessions sampled for these more phylogenetically distant species may be less accurate than among species with larger sampling like the other *Amaranthus* in this study (n = 6 to 120).

Notably, the weedy wild relative *A. hybridus* had F_ST_ values of 0.058 with *A. cruentus*, 0.128 with *A. caudatus*, and 0.224 with *A. hypochondriacus*, indicating its greater similarity with the first two species perhaps based on overlapping ecological zones. The other possible progenitor wild species, *A. powellii*, had similarity with *A. cruentus* (low F_ST_ value of 0.06) versus moderate distinction with *A. hypochondriacus* (0.169) and *A. hybridus* (0.164) and high distinction with *A. caudatus* (0.223) and *A. quitensis* (0.404).

### Clustering analysis

**Figure 2:**
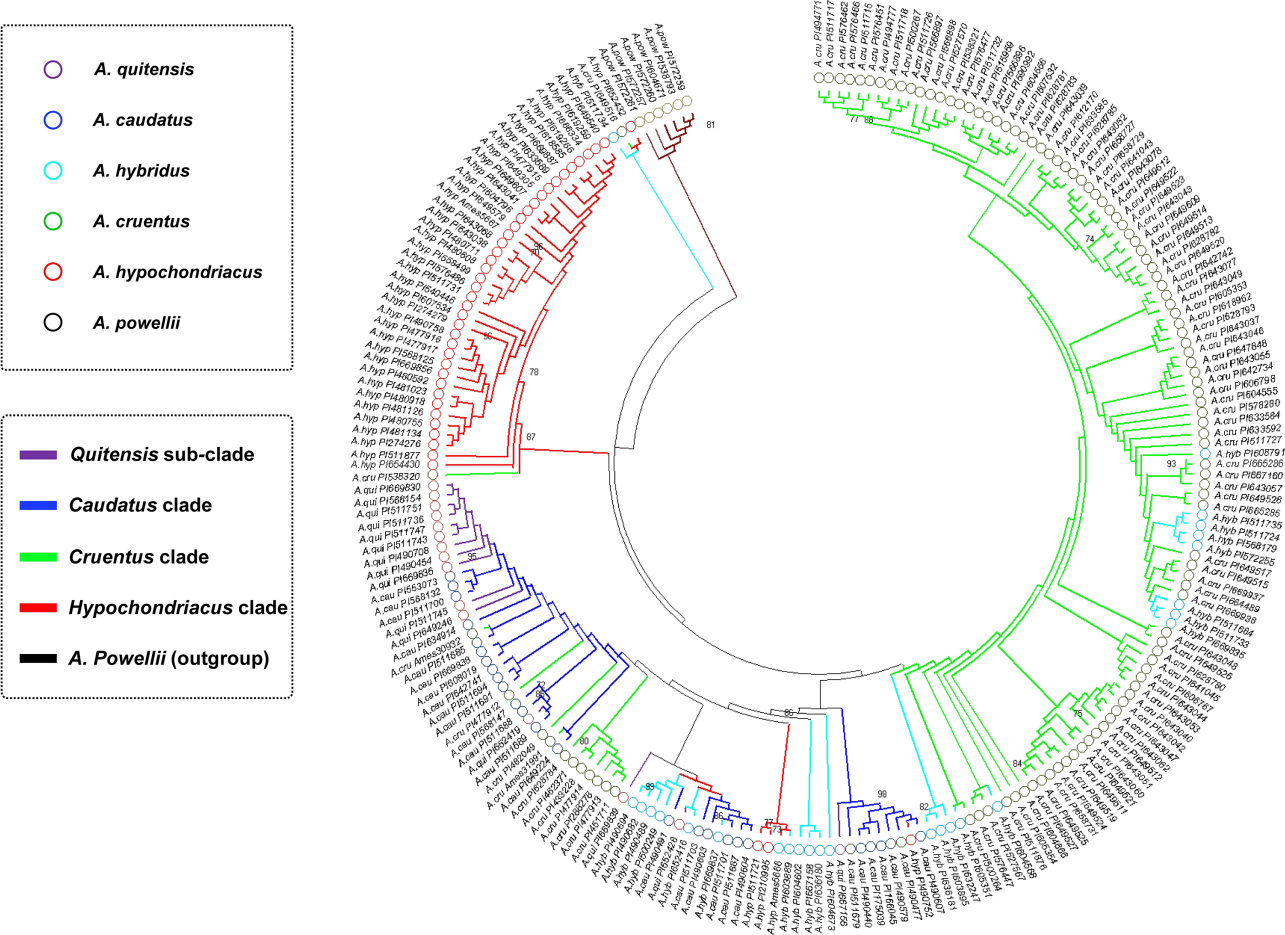
Clustering relationships between amaranth accessions based on maximum parsimony and color-coded by species from the *Amaranthus* genus and are abbreviated at the branch ends with plant introduction (PI) numbers provided. Numbers on key branches represent percent probability based on 1,000 bootstraps.

Relationships of species and accessions in each one were visualized with a dendrogram based on the 82 polymorphic SNP markers (**Figure 2**). The interpretation of the dendrogram verified the population structure analysis in terms of species relationships with groupings that could be divided into five clusters. A total of six genotypes made up cluster I, and all were *A. powellii*, with this group showing clear separation from the other five clades. This was an outgroup compared to the other groups with a bootstrap value of 64%.

Cluster II was made up of three genotypes, one each of *A. cruentus*, *A. hypochondriacus*, and *A. hybridus* supported at 63% bootstrap. Cluster III was all *A. hypochondriacus* with one-off type *A. cruentus* (PI58320) representing all Mesoamerican genotypes supported at 87%. The next two clusters were from the Andes region of South America: Cluster IV was made up of *A. quitensis* considered wild but with a few intervening genotypes supported at 50%. Cluster V had mostly *A. caudatus* but interspersed with a few *A. hybridus* possible relatives of progenitors for Andeans and some intervening Mesoamericans. Cluster VI was all made up of *A. cruentus* except for a few interspersed *A. hybridus* possible relatives or progenitors for this Mesoamerican species.

### Discriminant analysis for principal components

**Figure 3:**
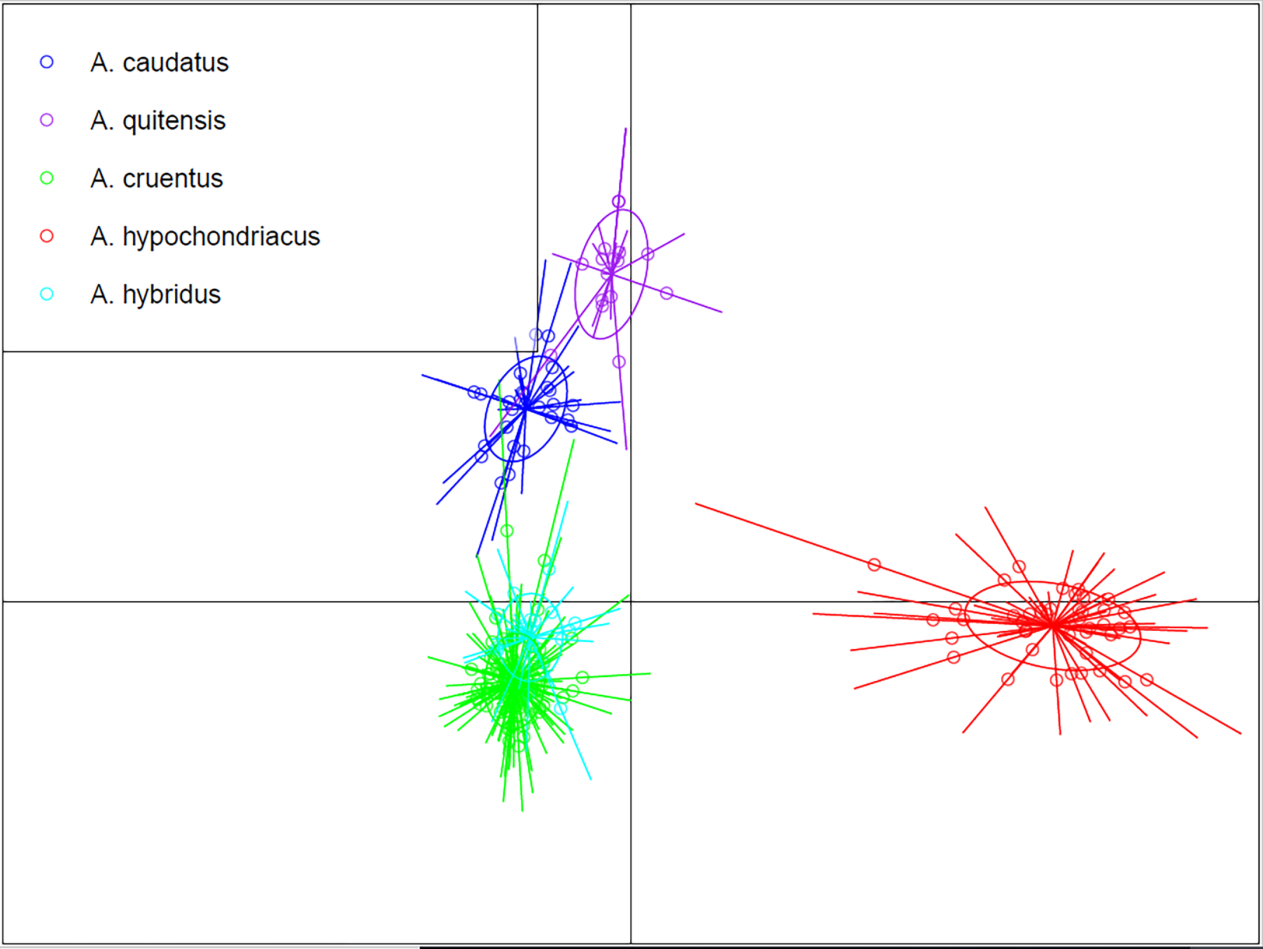
Discriminant analysis for principal components for five *Amaranthus* species.

To more fully understand the relationships of the five major species evaluated, we performed a DAPC, which reduces the intra-specific variation to better distinguish species-level differences (**Figure 3**). In that analysis, we found a clear separation of the five best-sampled species (n = 18 or above). The main cultivated grain amaranths were very separate, with *A. hypochondriacus* (red) very distant from *A. caudatus* (blue) and *A. cruentus* (green). The 24 samples of *A. hybridus* (light blue) clustered beneath *A. cruentus*. The 18 samples of *A. quitensis* clustered slightly above and to the right of *A. caudatus*. Since previous results with structure and with clustering analyses shown above suggested some admixture, it was revealed that DAPC could individualize the populations while still showing the previously observed similarity of Andeans cultivated and weedy (*A. caudatus* and *A. quitensis*) and the distance with Mesoamerican cultivated species. The location of *A. hybridus* beneath *A. cruentus* may suggest a tighter relationship with that species than others.

As a final analysis of species-level differences, we added the data of the possible progenitors and weed species of *Amaranthus* and performed a PCA in three-dimensional space (**Figure S1**) to gain another perspective on species separation. We found a clustering of *A. caudatus* separate from *A. quitensis* but in the same quadrant. *A. hypochondriacus* was at the maximum distance from these two species in a diagonal quadrant, while the majority of *A. cruentus* were at a moderate distance in a horizontal quadrant. Surprisingly, some *A. cruentus* could be grouped closely to *A. caudatus* and could be misidentified. The positions of *A. palmeri*, *A. powellii*, and *A. retroflexus* were difficult to assess, as they occupied space far from the latter two grain amaranths but closer to *A. hypochondriacus*. In the case of the five samples of *A. powellii*, they clustered tightly together, while the two samples of *A. retroflexus* were adjacent in the PCA plot. The single accession of *A. palmeri* was found below and distant from these two groups.

## Discussion

The population structure of grain amaranths and their immediate wild relatives in our proposed USDA core collection seems to be very pronounced and based on a geographic division of species between Vavilovian centers of origin. This was discoverable because the proposed core collection is representative of a diversity of cultivars and close relatives in North/Central and South America. In contrast, our previous studies have emphasized the possible originating species (Wu and Blair, 2017) using a subset of mostly wild and weedy; here, we have divided all grain types into groups into related clades as observed in the K = 2 population structure.

In the first clade of importance, the Andean species *A. caudatus* and *A. quitensis* are very closely related and perhaps reflect a weedy–cultivated complex that outcrosses each other and maintains constant introgression between the two species. The second larger clade in the core collection was the Mesoamerican with *A. cruentus* and *A. hypochondriacus* somewhat related but distinguishable with population structure at K − 3. At this same level of subpopulation division, the *A. hybridus* weed species has some genotypes related to the Andean clade and some to the Mesoamerican clade. This is logical, as *A. hybridus* is found across both continents of North and South America, while other wild precursor species *A. powellii* and *A. quitensis* are found on both sides of the division between continents. *A. quitensis* is more of a weedy relative than a wild one, confirming previous observations by Thapa et al. (Thapa and Blair, 2019; Thapa et al., 2021) and Wu and Blair (2017). The supplemental figure from Thapa et al. (2021) showed the geographic distribution of the species with collection site data and the diverse altitude and latitudes from which the accessions were sampled. A gap in collection sites is found geographically in Southern parts of Central America and Panama between the Andean and Mesoamerican groups, which is reinforced by the results found here.

The concept of separate gene pools for the south American region that includes the Andes Mountains compared to the Mesoamerican region of Central America and Mexico, which is less mountainous, is well established for wild versus cultivated and semi-domesticated chenopods (genus *Chenopodium*) including *Chenopodium quinoa*, or cultivated kinwa/quinoa from the Andes and *Chenopodium nuttalliae* from Mexico (Heiser and Nelson, 1974). Several New World legumes also have this pattern of Andean/Mesoamerican divergences, namely, *Phaseolus lunatus* (lima bean) and *Phaseolus vulgaris* (common bean) (Blair et al., 2009; Cortés et al., 2018).

However, unlike grain amaranths, these *Chenopodium* and *Phaseolus* spp. are more inter-crossable between gene pools and do not have different chromosome counts. Differences in genomic structure between different *Amaranthus* species might be the reason for crossing difficulties. For example, *A. cruentus* is thought to have a basic chromosome set of n = 17 versus n = 16 for *A. hypochondriacus*. Based on whole-genome sequencing (WGS), there are other differences (Deb et al., 2020); however, the same SNP markers function across both clades as we saw in this research. A better definition of the primary and secondary gene pools for each *Amaranthus* species is needed through crossing studies with a wide range of germplasm. Given the differences in chromosome counts between *A. caudatus* (1n = 17) and the Mesoamerican species (1n = 16) predicted by Andini et al. (2018), phenotyping studies trying to associate genetic markers like the ones we used here should always consider separating the two groups for more careful evaluation after any overall evaluation.

Another objective of this work was to establish the diversity within a proposed grain amaranth core collection, and this was confirmed by the dendrogram, PCA clustering along with AMOVA, and F_ST_ results for groups of accessions. The diversity values and overall structure compare favorably with previous studies of diversity within *Amaranthus* using SNP markers (Maughan et al., 2011; Thapa et al., 2021). In contrast, these studies used selected sets of markers to evaluate diversity, two genotyping by sequencing (GBS) studies (Stetter and Schmid, 2017; Wu and Blair, 2017) found similar results when evaluating random SNP. Later, Deb et al. (2020) used WGS to place six re-sequenced Indian cultivars in the dendrogram of genotypes studied by Wu and Blair (2017) and to compare their *A. cruentus* and *A. hypochondriacus* representatives to the reference genome from Lightfoot et al. (2017).

The question of geographical variation of grain amaranths is a valid one both within and between species since *A. caudatus* is found in a diverse homeland of inter-Andean valleys just as the center of origin of *A. cruentus* and *A. hypochondriacus* consists of lowlands to highlands and dry savannah zones to flanks of the central Mexican volcanos. The F_ST_ values for these three species plus *A. hybridus* and *A. quitensis* are shown in **Figure S2A**. Higher F_ST_ values (0.25 to 0.4) occurred within comparisons of *A. hypochondriacus* accessions, then within species for the two wild relatives, and then *A. caudatus* (0.1 to 0.3). *A. cruentus* accessions had significantly lower F_ST_ values than within the *A. hypochondriacus* species. A heat map (**Figure S2B**) of F_ST_ values shows a relationship among the five species concordant with previously discussed population structure and PCA results for our CC accessions with values of 0 to 1 where high F_ST_ values implied larger degrees of differentiation between Andean and Mesoamerican species.

A further result from our study was the validation of a proposed CC for *Amaranthus* species and accessions of interest to crop breeders. The full collection for the genus at the USDA consists of 3,383 accessions in Ames, Iowa, of which currently 3,231 are available for distribution. However, only 2,899 accessions have site collection data, which is often a requirement for establishing a “core”. In addition, many accessions are breeding lines from the Rodale research program of the 1970s to 1990s, which would not represent raw germplasm typical of the overall gene bank. Furthermore, weedy types versus those used as vegetables or grain sources are difficult to parse in the collection since when collecting *in situ*, some phenotypic parameters of each species can be misidentified. Therefore, our group of 260 genotypes does represent an approximate 10% core for the germplasm at USDA that falls into the categories of interest as Andean or Mesoamerican grain species and relatives.

Overall, the proposed USDA core collection for grain amaranths is adequate for covering the diversity of cultivated grain species for both Andean and Mesoamerican centers of origin as well as that in the wild relative *A. hybridus*. As this same CC has been partially evaluated for a larger number of SNPs (Wu and Blair, 2017), we feel that this collection could be a “temperate zone” core useful for genome-wide association study (GWAS) evaluations of physiological traits.

Furthermore, the collection presented here complements a CC made for vegetable species of amaranths (*Amaranthus tricolor*, for example) by Hoshikawa et al. (2023). However, a decision should be made for the grain CC whether to include wild species *A. hybridus* and *A. quitensis*, as these tend to be hard to evaluate for yield traits, having smaller dark seeds and shattering before maturity. If these were removed along with *A. powellii* and *A. retroflexus*, there would still be enough representation at n = 197 for the presented core to be useful for GWAS specifically in grain types. Given recent results on the comparative physiology of the grain amaranths where *A. cruentus* was highly drought tolerant (Netshimbupfe et al., 2022), our larger sampling of *A. cruentus* at n = 120 compared to *A. caudatus* (n = 33) and *A. hypochondriacus* (n = 44) seems appropriate.

As our number of SNP marker number was limited (but highly polymorphic), we recommend re-sequencing with PacBio or Illumina skimSeq but using multiple reference genomes for more SNP discovery. Previous studies for SNPs (Maughan et al., 2011, Stetter and Schmid, 2017, Wu and Blair, 2017; Thapa et al., 2021) referenced the Plainsman variety, which is possibly of mixed Mesoamerican ancestry (Lightfoot et al., 2017; Deb et al., 2020). Maughan et al. (2009), in contrast, emphasized *A. caudatus* for genomics. We recommend that further study with KASP, GBS, or re-sequencing be performed to define the limits and extents of the primary, secondary, and tertiary gene pools for each of the *Amaranthus* species, but the concept presented here of separating gene pools by the center of origin is useful to grain amaranth researchers.

## Data availability statement

The original contributions presented in the study are included in the article/**Supplementary Material**. Further inquiries can be directed to the corresponding author.

## Author contributions

MB supervised the study and wrote the manuscript. MB, DB, and XW conceived the different parts of this study especially in design, germplasm selection, and laboratory execution, respectively. JL and MB-B, as visiting students to the laboratory of MB, carried out the marker genotyping with assistance from XW. Funding for the study was obtained by MB. Germplasm was provided by DB and his team. JL and MB-B organized data collection and prepared figures and tables with the help of MB. All authors helped interpret data, and DB along with JL made edits to the manuscript prepared by MB. All authors contributed to the article and approved the submitted version.

## Supplementary material

Supplementary Figure 1 Principal component Analysis (PCA) of the eight species of *Amaranthus* considered in this study, highlighting the grain amaranths *A. caudatus* in dark blue, *A. cruentus* in dark green and *A. hypochondriacus* in red.

Supplementary Figure 2 F_ST_ values at the population level for well sampled species (N ≥18 accessions) showing a. Boxplot for F_ST_ variation within the five species. b. Heatmap plot for F_ST_ variation between the five species.
