# Supplementary figures and images for: Differentiation of Andean and Mesoamerican accessions in a proposed core collection of grain amaranths
Source: Front Plant Sci. 2023 Mar 22;14:1144681. doi: 10.3389/fpls.2023.1144681 (PMC10073572; doi:10.3389/fpls.2023.1144681)

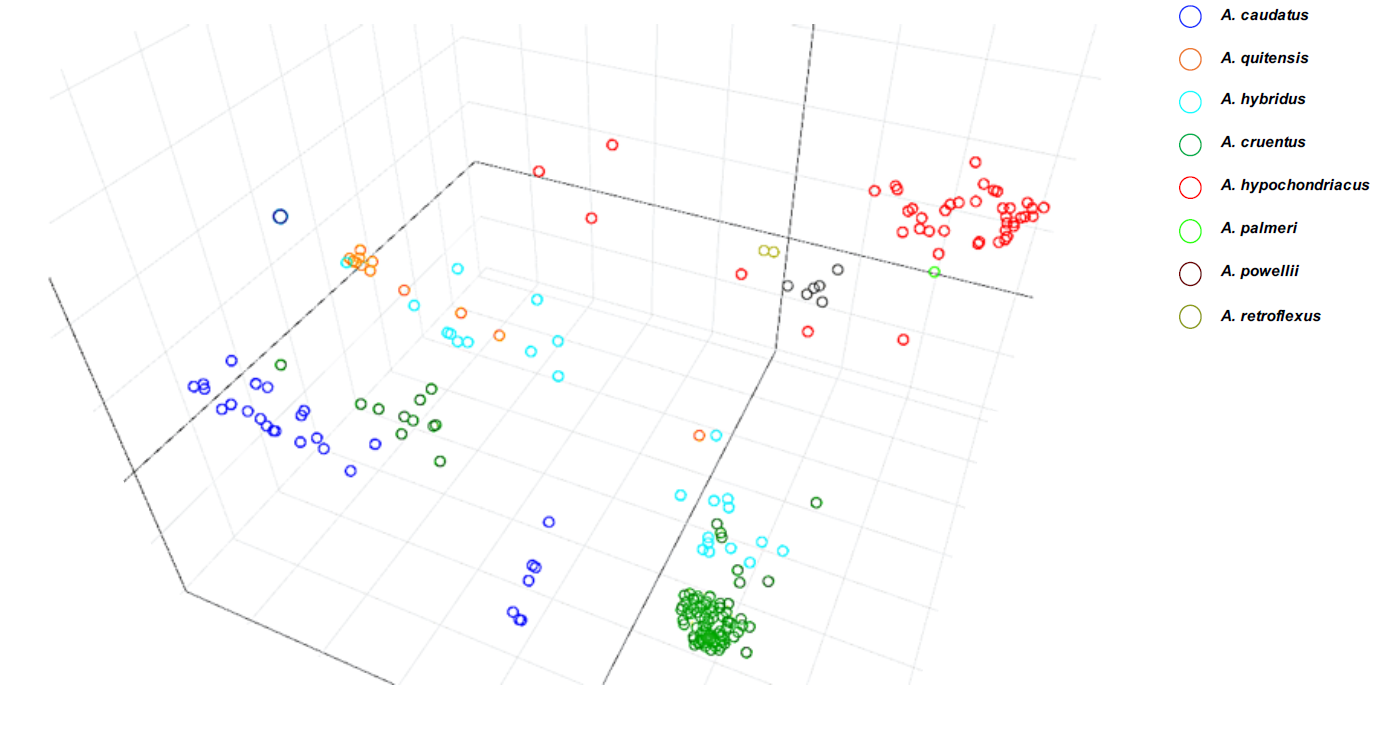

Supplement: Supplementary Figure 1 — Principal component Analysis (PCA) of the eight species of Amaranthus considered in this study, highlighting the grain amaranths A. caudatus in dark blue, A. cruentus in dark green and A. hypochondriacus in red. [file Image_1.tif]

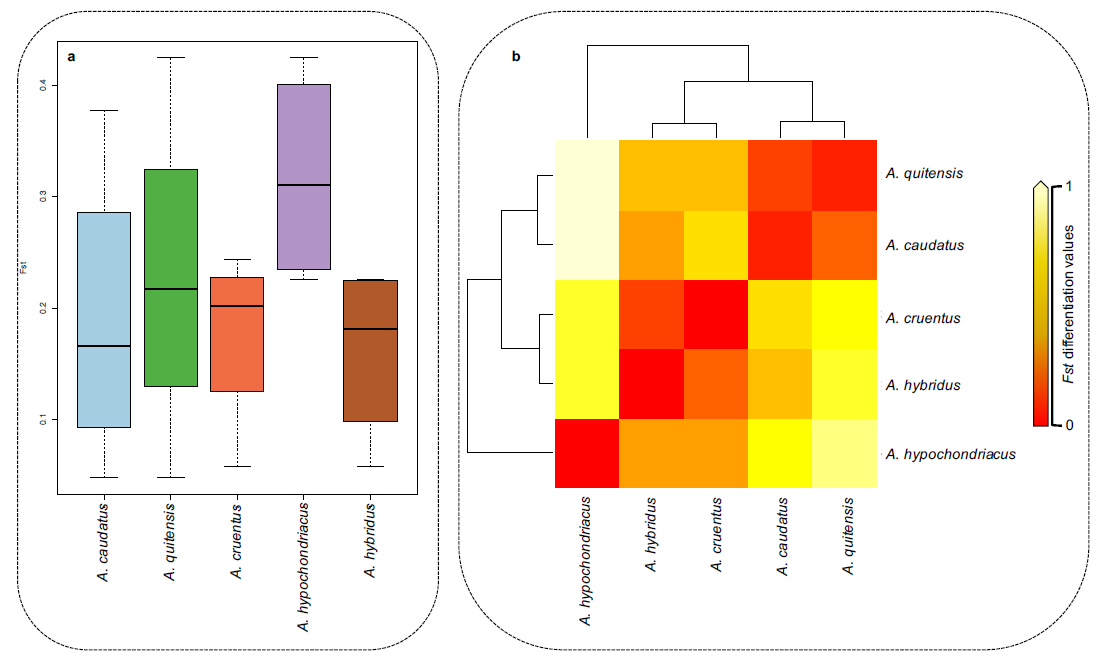

Supplement: Supplementary Figure 2 — FST values at the population level for well sampled species (N ≥18 accessions) showing a. Boxplot for FST variation within the five species. b. Heatmap plot for FST variation between the five species. [file Image_2.tif]
