# Supplementary material for: Differentiation of Andean and Mesoamerican accessions in a proposed core collection of grain amaranths
Source: Front Plant Sci. 2023 Mar 22;14:1144681. doi: 10.3389/fpls.2023.1144681 (PMC10073572; doi:10.3389/fpls.2023.1144681)
Supplement: Supplementary file 3 [file Table_1.docx]

**Supplemental Table 1.** Genetic diversity for 82 SNP markers evaluated on the core collection of grain amaranth accessions.

| **SNP**  **no.** | **Amaranth**  **SNP Name** | **Major Allele Freq (MAF)** | **Genetic diversity (GD)** | **Observed**  **Heterozygosity (H_o_)** | **PIC**  **value** |
| --- | --- | --- | --- | --- | --- |
| 1 | AM17977 | 0.61 | 0.51 | 0.35 | 0.42 |
| 2 | AM18039 | 0.71 | 0.42 | 0.01 | 0.35 |
| 3 | AM19011 | 0.72 | 0.41 | 0 | 0.33 |
| 4 | AM19559 | 0.86 | 0.24 | 0.25 | 0.21 |
| 5 | AM19643 | 0.72 | 0.44 | 0.22 | 0.4 |
| 6 | AM19746 | 0.52 | 0.52 | 0.18 | 0.4 |
| 7 | AM20177 | 0.8 | 0.32 | 0.11 | 0.27 |
| 8 | AM21724 | 0.78 | 0.35 | 0.12 | 0.29 |
| 9 | AM21859 | 0.52 | 0.61 | 0 | 0.54 |
| 10 | AM22137 | 0.73 | 0.4 | 0 | 0.32 |
| 11 | AM22892 | 0.79 | 0.33 | 0.12 | 0.28 |
| 12 | AM23006 | 0.66 | 0.45 | 0.01 | 0.35 |
| 13 | AM23128 | 0.72 | 0.4 | 0.01 | 0.32 |
| 14 | AM23196 | 0.81 | 0.32 | 0.03 | 0.27 |
| 15 | AM23262 | 0.61 | 0.55 | 0.01 | 0.49 |
| 16 | AM24029 | 0.72 | 0.4 | 0 | 0.33 |
| 17 | AM24210 | 0.45 | 0.65 | 0 | 0.57 |
| 18 | AM24401 | 0.72 | 0.4 | 0 | 0.32 |
| 19 | AM27616 | 0.72 | 0.41 | 0 | 0.34 |
| 20 | AM27642 | 0.49 | 0.63 | 0 | 0.55 |
| 21 | AM17870 | 0.58 | 0.5 | 0.32 | 0.38 |
| 22 | AM18185 | 0.75 | 0.38 | 0.04 | 0.31 |
| 23 | AM19378 | 0.55 | 0.49 | 0.69 | 0.37 |
| 24 | AM19426 | 0.64 | 0.47 | 0.7 | 0.37 |
| 25 | AM19707 | 0.63 | 0.53 | 0 | 0.47 |
| 26 | AM19842 | 0.72 | 0.41 | 0.06 | 0.33 |
| 27 | AM19855 | 0.5 | 0.6 | 0 | 0.52 |
| 28 | AM20180 | 0.73 | 0.4 | 0.01 | 0.32 |
| 29 | AM20403 | 0.53 | 0.51 | 0.1 | 0.4 |
| 30 | AM20533 | 0.87 | 0.23 | 0.27 | 0.2 |
| 31 | AM21310 | 0.75 | 0.38 | 0 | 0.31 |
| 32 | AM21336 | 0.83 | 0.29 | 0.33 | 0.25 |
| 33 | AM21842 | 0.67 | 0.48 | 0.16 | 0.42 |
| 34 | AM22476 | 0.75 | 0.38 | 0 | 0.31 |
| 35 | AM22487 | 0.67 | 0.45 | 0.24 | 0.37 |
| 36 | AM22649 | 0.63 | 0.47 | 0.74 | 0.36 |
| 37 | AM23703 | 0.56 | 0.5 | 0.01 | 0.38 |
| 38 | AM24078 | 0.78 | 0.35 | 0 | 0.29 |
| 39 | AM24266 | 0.84 | 0.27 | 0 | 0.24 |
| 40 | AM24531 | 0.7 | 0.44 | 0.06 | 0.37 |
| 41 | AM24819 | 0.65 | 0.46 | 0.29 | 0.36 |
| 42 | AM26171 | 0.72 | 0.41 | 0 | 0.34 |
| 43 | AM27610 | 0.7 | 0.42 | 0.01 | 0.33 |
| 44 | AM27626 | 0.69 | 0.43 | 0.04 | 0.34 |
| 45 | AM18245 | 0.61 | 0.48 | 0.03 | 0.36 |
| 46 | AM18741 | 0.79 | 0.33 | 0.02 | 0.28 |
| 47 | AM19210 | 0.69 | 0.43 | 0.61 | 0.34 |
| 48 | AM19501 | 0.86 | 0.25 | 0 | 0.22 |
| 49 | AM19534 | 0.82 | 0.3 | 0 | 0.25 |
| 50 | AM19834 | 0.79 | 0.33 | 0.02 | 0.27 |
| 51 | AM19963 | 0.96 | 0.08 | 0.01 | 0.08 |
| 52 | AM20065 | 0.88 | 0.21 | 0.03 | 0.19 |
| 53 | AM20178 | 0.59 | 0.48 | 0 | 0.37 |
| 54 | AM20333 | 0.85 | 0.25 | 0 | 0.22 |
| 55 | AM20370 | 0.94 | 0.11 | 0.11 | 0.1 |
| 56 | AM20790 | 0.9 | 0.18 | 0.02 | 0.17 |
| 57 | AM20885 | 0.77 | 0.35 | 0 | 0.29 |
| 58 | AM21250 | 0.81 | 0.31 | 0 | 0.26 |
| 59 | AM21275 | 0.87 | 0.23 | 0.04 | 0.2 |
| 60 | AM21331 | 0.63 | 0.46 | 0 | 0.36 |
| 61 | AM22029 | 0.55 | 0.49 | 0 | 0.37 |
| 62 | AM22416 | 0.81 | 0.31 | 0 | 0.26 |
| 63 | AM23934 | 0.79 | 0.33 | 0.01 | 0.28 |
| 64 | AM24020 | 0.88 | 0.22 | 0 | 0.19 |
| 65 | AM24104 | 0.93 | 0.14 | 0.07 | 0.13 |
| 66 | AM24152 | 0.81 | 0.3 | 0 | 0.26 |
| 67 | AM24457 | 0.67 | 0.44 | 0.02 | 0.34 |
| 68 | AM24490 | 0.82 | 0.3 | 0.01 | 0.25 |
| 69 | AM24501 | 0.63 | 0.47 | 0 | 0.36 |
| 70 | AM24909 | 0.82 | 0.29 | 0.02 | 0.25 |
| 71 | AM24924 | 0.86 | 0.24 | 0.01 | 0.21 |
| 72 | AM25418 | 0.95 | 0.09 | 0.02 | 0.09 |
| 73 | AM25548 | 0.79 | 0.33 | 0.01 | 0.28 |
| 74 | AM25606 | 0.91 | 0.16 | 0.01 | 0.15 |
| 75 | AM26133 | 0.8 | 0.32 | 0 | 0.27 |
| 76 | AM27605 | 0.84 | 0.26 | 0.02 | 0.23 |
| 77 | AM27611 | 0.77 | 0.35 | 0.03 | 0.29 |
| 78 | AM27630 | 0.84 | 0.26 | 0 | 0.23 |
| 79 | AM27632 | 0.54 | 0.5 | 0.01 | 0.37 |
| 80 | AM27634 | 0.8 | 0.32 | 0 | 0.27 |
| 81 | AM27636 | 0.51 | 0.5 | 0.72 | 0.37 |
| 82 | AM27653 | 0.58 | 0.49 | 0.03 | 0.37 |
|  | Mean | 0.73 | 0.38 | 0.09 | 0.31 |

Abbreviations: MAF, Major Allele Frequency; GD, Genetic diversity; H_o_, Observed Heterozygosity; PIC, Polymorphism Information Content.
